# Supplementary material for: Persistent warm-eddy transport to Antarctic ice shelves driven by enhanced summer westerlies
Source: Nat Commun. 2024 Jan 22;15:671. doi: 10.1038/s41467-024-45010-x (PMC10803808; doi:10.1038/s41467-024-45010-x)
Supplement: Supplementary file 1 — Supplementary Information [file 41467_2024_45010_MOESM1_ESM.pdf]

Persistent warm-eddy transport to Antarctic ice shelves driven by enhanced summer westerlies

Supplementary Figures

There are 10 additional supplementary figures supporting the main article.

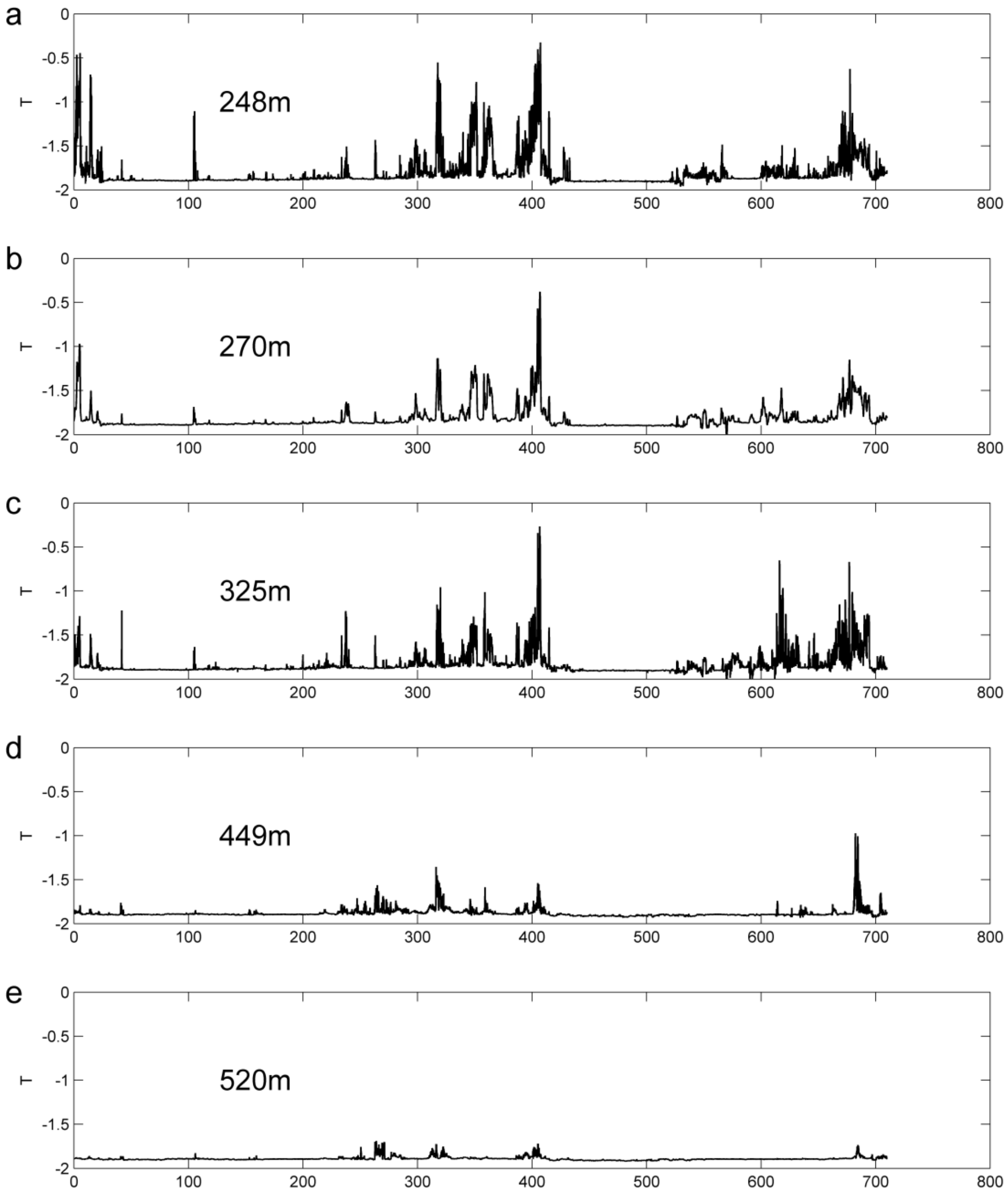

Supplementary Fig. 1 | Hourly potential temperature in different levels of mooring. (a) 248m. **(b)** 270m. **(c)** 325m. **(d)** 449m. **(e)** 520m. X-axis is days since 1-March-2013.

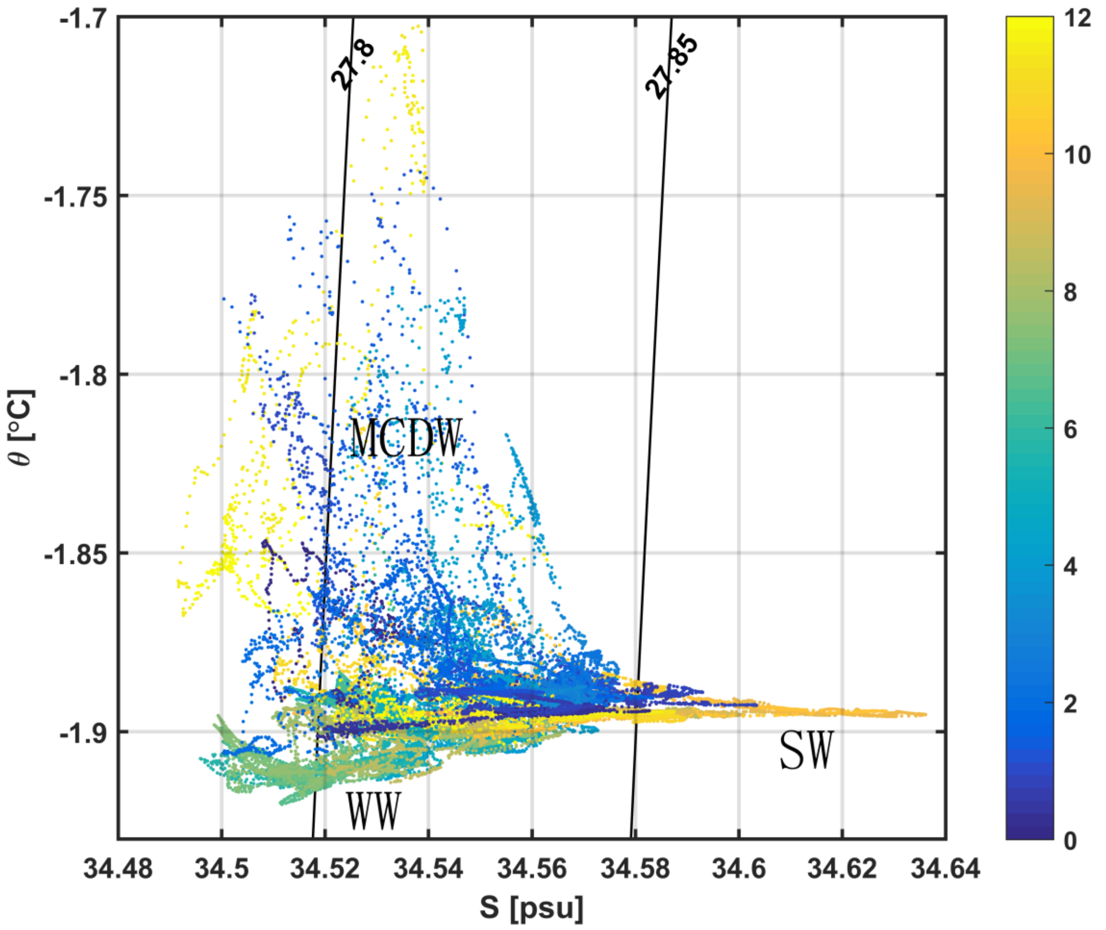

Supplementary Fig. 2 | Figure S6. T-S diagram of the 520m observations on the mooring during 2013-2015. Colors represent calendar month. Black lines are the potential density surface.

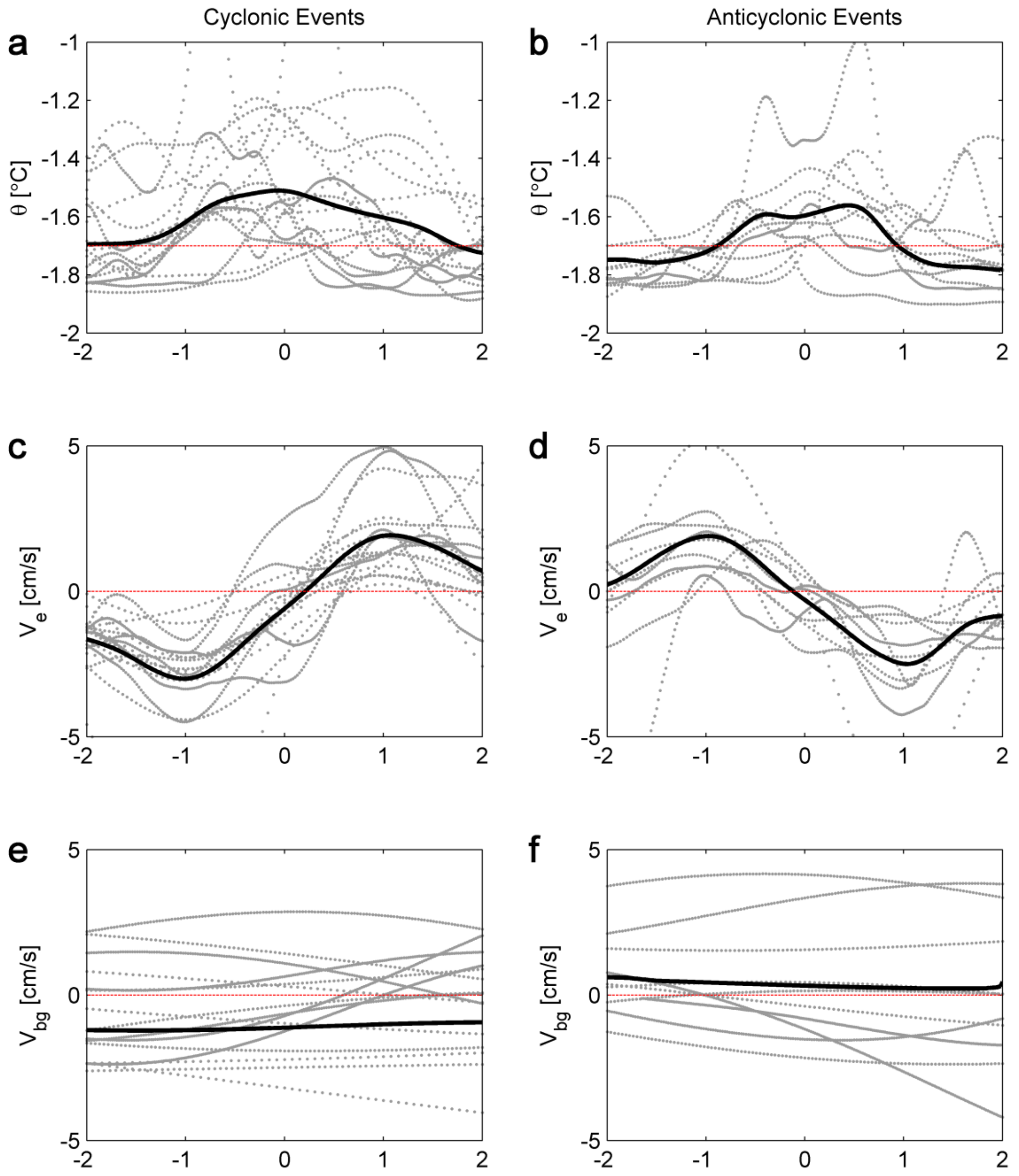

Supplementary Fig. 3 | Composition analysis of eddies at 270m. (a) and **(b)** potential temperature (red dashed lines show -1.7 ºC position); **(c)** and **(d)** cross-stream velocity; **(e)** and **(f)** meridional component of background velocity. The sample mean is given in bold black line. The time is normalized using equation (2).

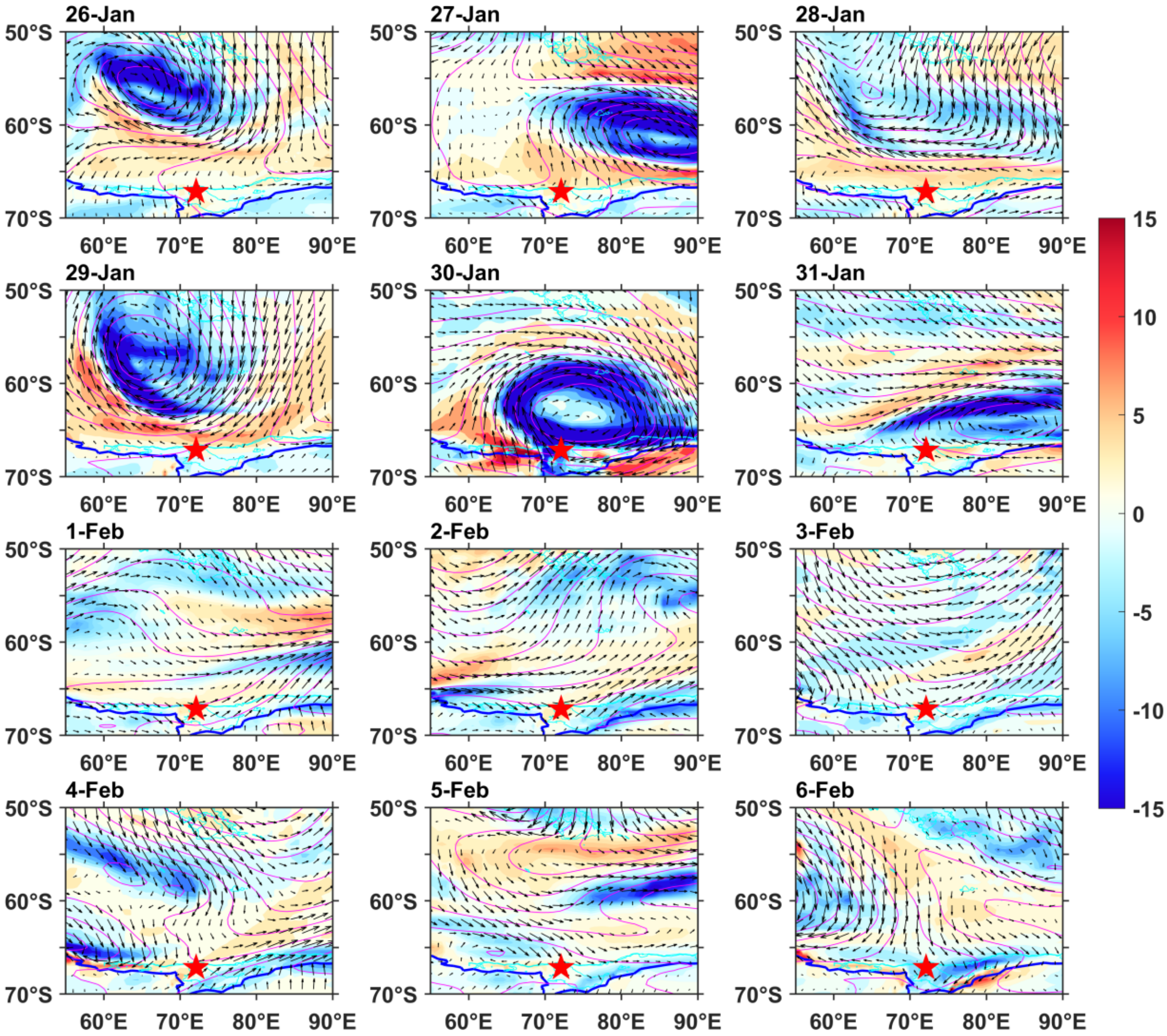

Supplementary Fig. 4 | Evolution of wind forcing anomaly during 26-January~6-February in 2013. (wind stress curl anomaly, shade, unit: 10^{-10} m • s^{-2}; wind anomaly, arrows; Sea Level Pressure anomaly, contours).

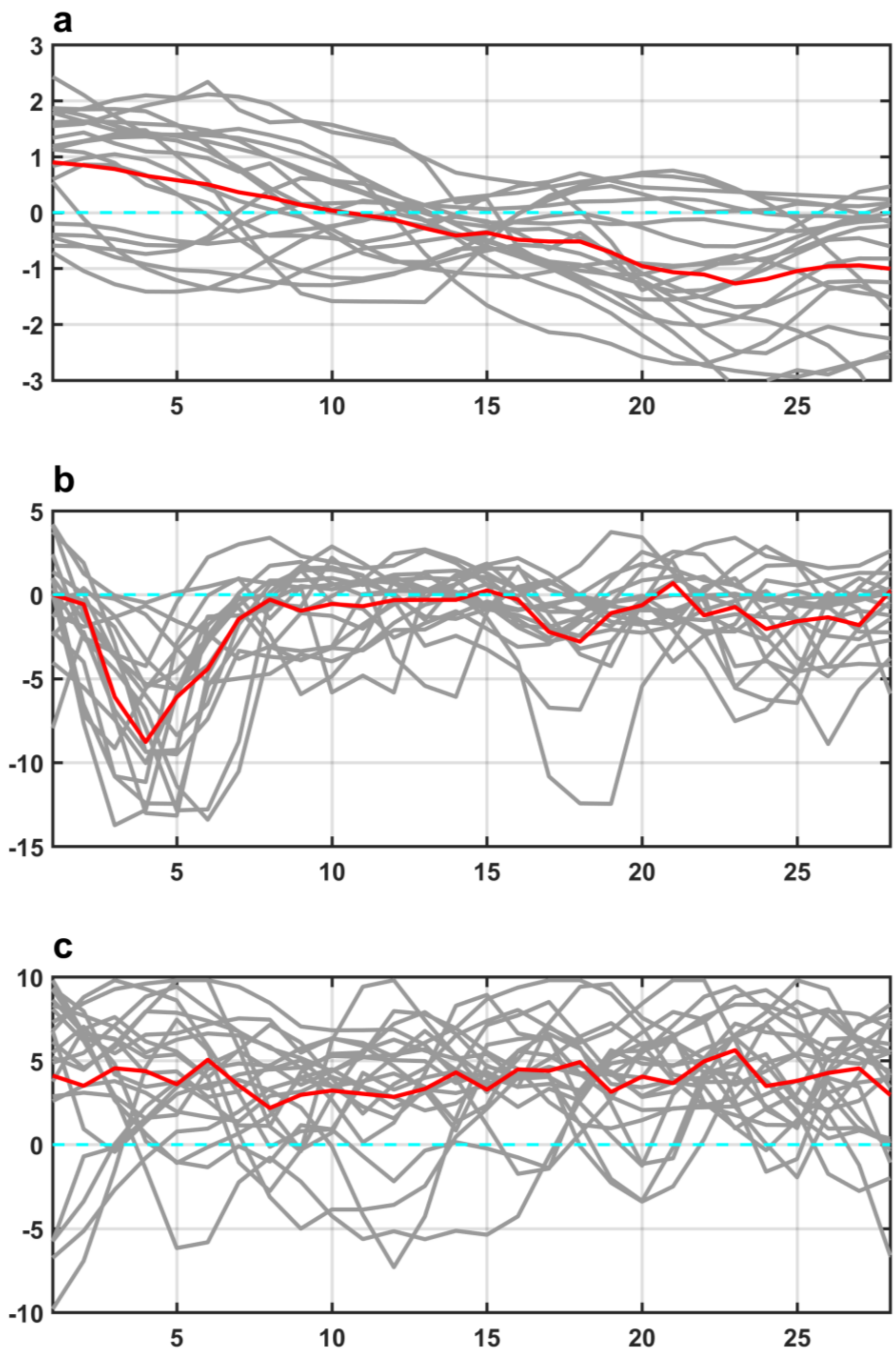

Supplementary Fig. 5 | Composition analysis of all warm events. a, Evolution of SSH anomaly (averaged in the 70-74°E, 65.7-67.1^{o}S region, black line, unit: cm). **b,** Evolution of wind stress curl anomaly (averaged in the 65-85°E, 56-66°S region, red line, unit: 2×10^{-10} m • s^{-2}). **c,** Evolution of zonal wind anomaly (averaged in the 65-85°E, 56-66°S region, cyan line, unit: 3 m • s^{-1}). Red lines are the ensemble mean. X-axis is days.

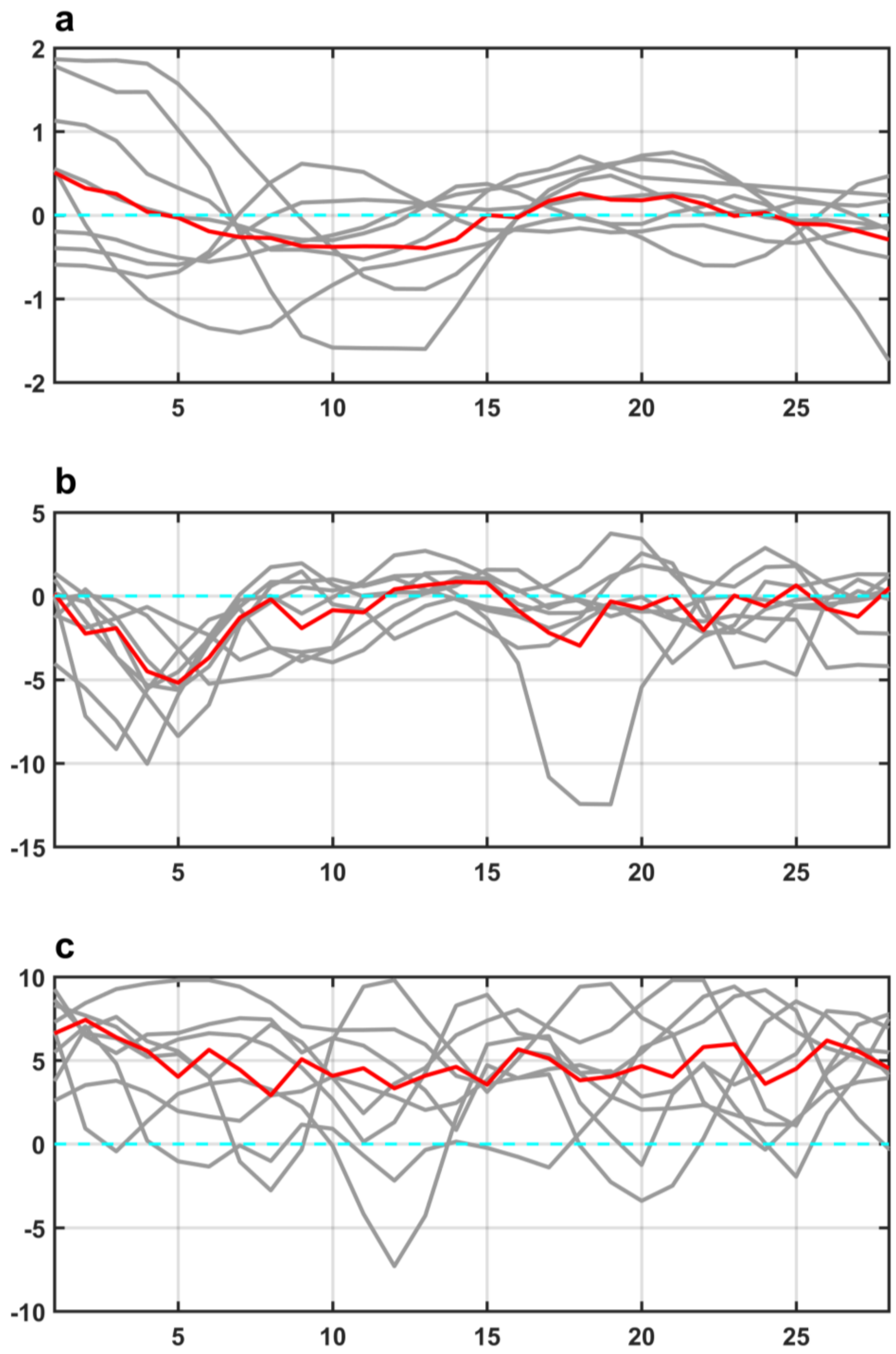

Supplementary Fig. 6 | Composition analysis of warm events with anticyclonic eddies. a, Evolution of SSH anomaly (averaged in the 70-74ºE, 65.7-67.1ºS region, black line, unit: cm). **b,** Evolution of wind stress curl anomaly (averaged in the 65-85ºE, 56-66ºS region, red line, unit: 2×10^{-10} m • s^{-2}). **c,** Evolution of zonal wind anomaly (averaged in the 65-85ºE, 56-66ºS region, cyan line, unit: 3 m •s^{-1}). Red lines are the ensemble mean. X-axis is days.

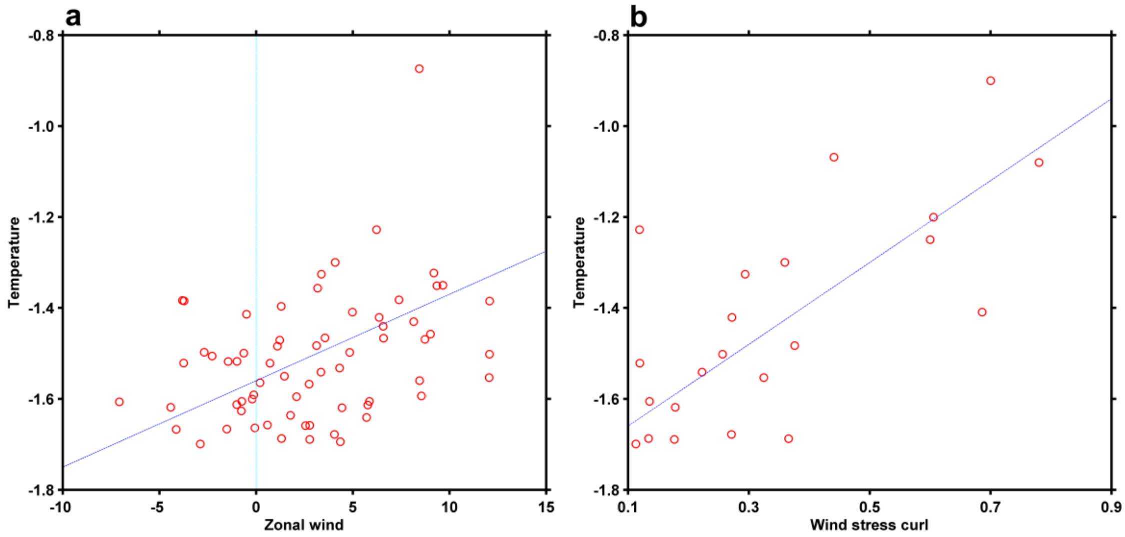

Supplementary Fig. 7 | Diagrams of warm signals to zonal wind and wind stress curl. a, Warm signals VS. Zonal wind (unit: m • s^{-1}) in (68°E-78°E, 60°S-67°S) region. Wind leads warm events for 4-8 days. **b**, Warm signals VS. Cyclonic wind stress curl (unit: 10^{-10}m • s^{-2}) in (66°E-78°E, 65°S-68°S) region. Wind stress curl leads warm events for 4-8 days. The regression is shown in blue dashed line.

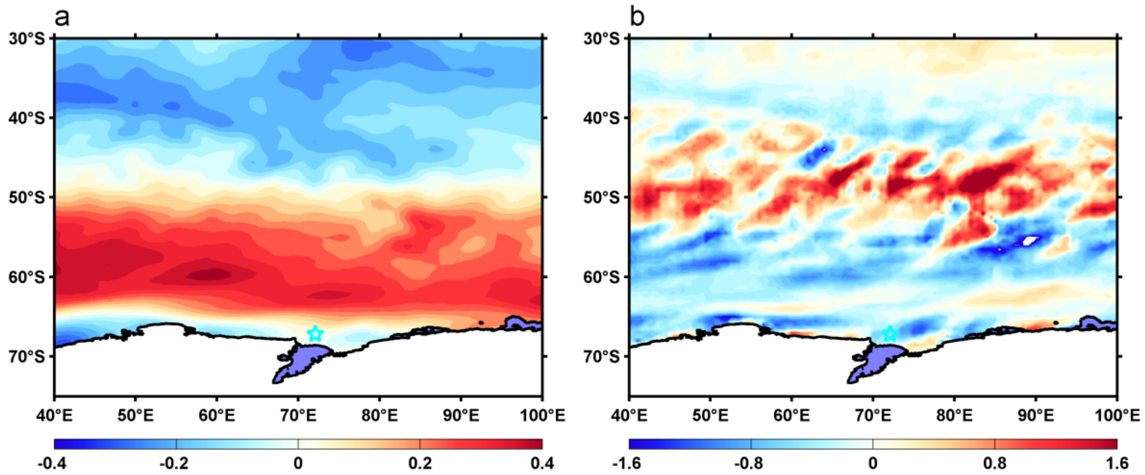

Supplementary Fig. 8 | Austral summer (DJF) wind trend during 1979-2018 period. a, Zonal wind trend (m • s^{-1}/(10years)). **b,** Wind stress curl trend (10^{-10}m • s^{-2}/(10years)). The mooring location is shown with a cyan star.

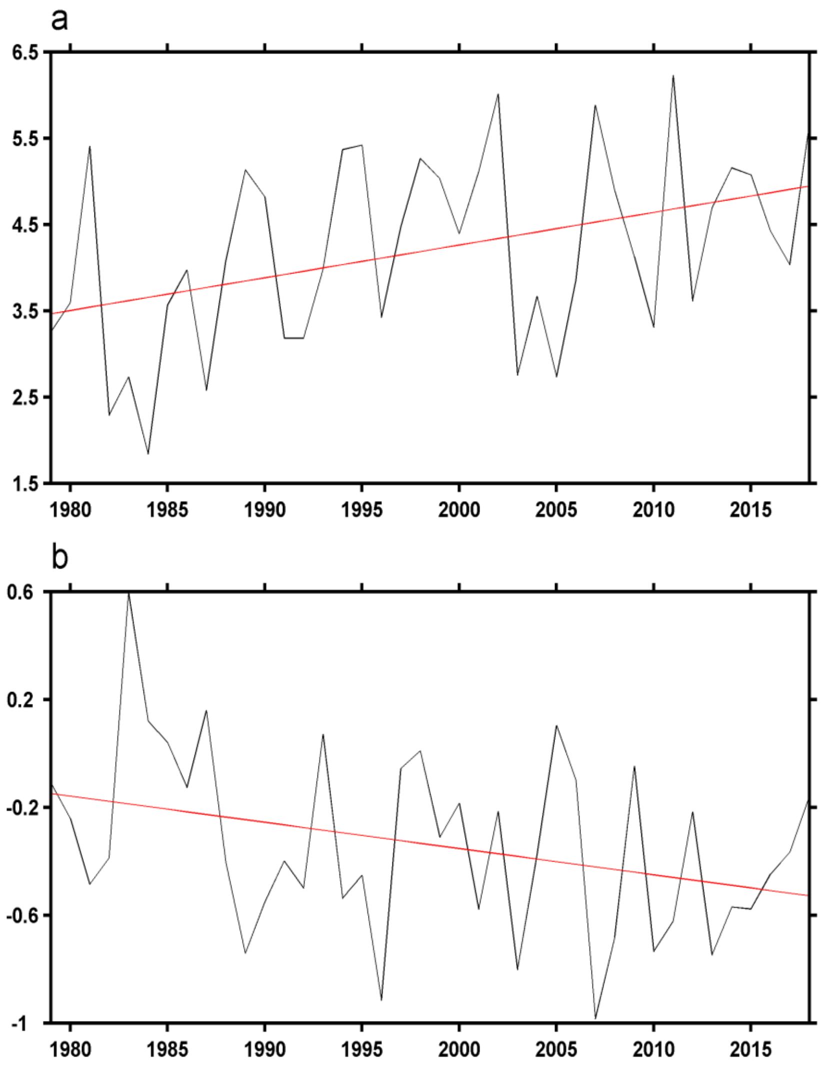

Supplementary Fig. 9 | Variation of austral summer (DJF) wind during 1979-2018 period. a, Zonal wind (m • s^{-1}) in (40E-100E, 55S-63S) region. **b,** Wind stress curl (10^{-10}m • s^{-2}) in (65E-78E, 65S-68S) region.

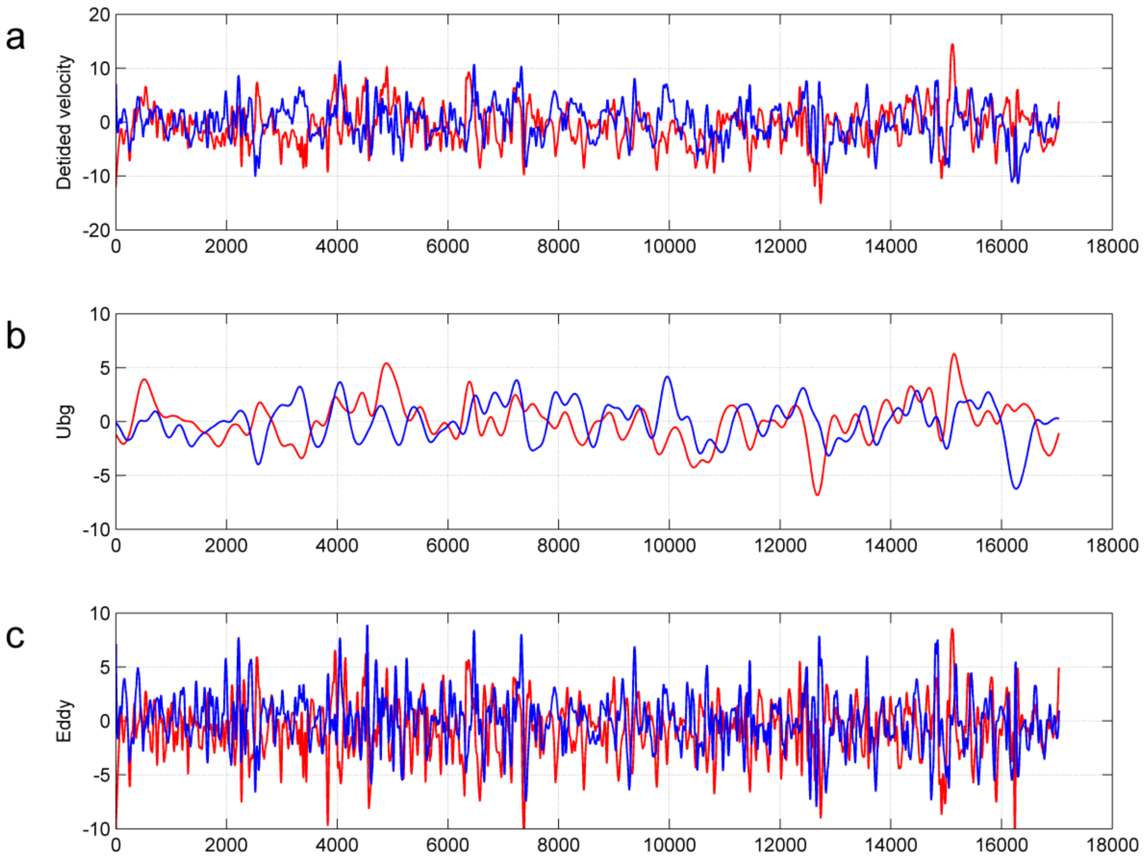

Supplementary Fig. 10 | Hourly u（red）and v (blue) components at 270m. a, de-tided velocity. **b,** background velocity (the first component of low-pass filtered with 20 hours). **c,** eddy velocity. Unit: cm/s. X-axis is hours since 1-March-2013.
